# Nitrergic Response TO Cyclophosphamide Treatment in Blood and Bone Marrow

**DOI:** 10.2174/1874091X00802010081

**Published:** 2008-06-03

**Authors:** G.A Kevorkian, N.Kh Alchujyan, N.H Movsesyan, H.L Hayrapetyan, A.G Guevorkian, R.M Ohanyan, S.S Dagbashyan

**Affiliations:** H. Buniatian Institute of Biochemistry NAS RA, 5/1 P.Sevak St., 0014, Yerevan, Republic of Armenia

**Keywords:** Cyclophosphamide, arginine, blood formed elements, citrulline, nitric oxide synthase, marrow, rat

## Abstract

Daily intraperitoneal injection of cyclophosphamide (CPA) (50 mg∙kg^-1^ of body weight) for 5 days resulted in reduced levels of marrow and blood cellularity, which was most pronounced in 18 days post-treatment (*pt*). On day 18 after CPA treatment the enhancedlevels of nitric oxide (NO) precursors and metabolites (L-arginine, L-citrulline, reactive nitrogen species (RNS)) of marrow and blood cells (platelet, neutrophil, lymphocyte and monocyte) resulted from up-regulation of Ca(II)/calmodulin(CaM)-independent “inducible” NO synthase (iNOS), with a lessercontribution of Ca(II)/CaM-dependent “constitutive” cNOS isoforms to systemic NO.**Biphasic response to CPA of marrow nitrergic system, i.e. both iNOS and cNOS showed significantly depressed activities, as well as diminished levels of NO metabolites on day 9 *pt*, suggested that signals in addition to NO might be involved in CPA-induced inhibition of hematopoesis, while a gradual increase of neutrophil and platelet NOS activity appeared to be contributed to a CPA-induced development of granulopenia, thrombocytopenia and hemorrhage.

## INTRODUCTION 

Accumulating data suggest that an immunosuppressive drug cyclophosphamide (CPA) widely used in chemotherapymediates immunosuppressive and tumoricidal effects *via *nitric oxide (NO) and its reactive intermediates [reviewed in Ref. 1, 2]. NO controls expression of a wide range of anti-proliferative genes through diverse signaling pathways [3, 4]. Inhibition of the activation of nuclear transcription factor-κB (NF-κB) in antigen-presenting cells is one of the molecular mechanisms of immunosuppressive drugs. Morphine mediates NO release in naloxone antagonized manner in monocytes and neutrophils and thereby affects NF-κB nuclear binding in these cells and a subsequent expression of pro-inflammatory cytokines or adhesion molecules**[5].**NO is generated from L-arginine by a family NOS enzymes: the most known Ca(II)/ calmodulin (CaM) -dependent “constitutive” NOS isoforms (cNOS), neuronal NOS (nNOS) and endothelial NOS (eNOS), that require CaM for activation and depend on the level of intracellular calcium to sustain bonding of CaM to their oxygenase domains, and Ca(II)/CaM-independent “inducible” NOS (iNOS), an effector molecule of the innate immune system, a regulatory component of host defense against pathogens and inflammation [ reviewed in Ref.^ 6^,^ 7^]. Distinct NOSs have unique roles in mediating physiological responses or in responding to disease, and now shouldalso be considered in the design of NO-related therapeutic targets**[reviewed in Ref.^ 8^,^ 9^]. cNOS-derived NO stabilizes the IκB-α-NF-κB inhibitory complex, reducing the release of pro-inflammatory mediators (the effectors of NF-κB activation: TNF-α, IL-1β, IL-2, 6, -8) – a process that is counteracted by iNOS-NO [10]. Therapy with CPA, which has a direct myelo-suppressive effect, has been related to a risk of developing cytopenia, leucopenia etc., associated with immune systems damage including nitrergic function. In this context, this study was performed to ascertain the CPA effect on the pharmacologically and functionally distinguished NOS isoforms. Here we report our initial observations on the NO generating system of blood and bone marrow in rat model of CPA-induced inhibition of hematopoiesis accompanied with granulopenia, thrombocytopenia and aplasia, as well.

## MATERIALS AND METHODOLOGY

N^G^-monomethyl-L-arginine (NMMA) was obtained from Calbiochem (La Jolla, CA). All other reagents were purchased from Sigma Chemical Co (St Louis, MO).

### Animal Care and Immunosuppression Procedure

All procedures involving animals were approved by the respectiveInstitutional Animal Care and Ethics Committee of the National Academy of Sciences of the Republic of Armenia. Forall experiments, groups (*n* = 6/group) of adult female rats weighing 200–250 g were used. Animals were housed at a constanttemperature and humidity, andhad unrestricted access to a standard diet and tap water. Cyclophosphamide (50 mg∙kg^-1^ of body weight) reconstituted in sterile distilled water was administered *via *intraperitoneal (i.p.) injection (500 μl) for 5 days. To confirm the duration of cytopenia, differential counts of blood and marrow samples were carried out, which showed that most of the tested rats remained cytopenic for up to 18 days post-treatment (*pt*). Animals were divided into groups: one group served as control and the other two as CPA-treated groups, in which rats were sacrificed by decapitation 9 days and 18 days after the last CPA administration.

### Isolation of Blood Formed Elements and Marrow

Blood samples obtained at time of death were dissolved in 4 mM disodium-EDTA in a 9:1 ratio, mixed with 6% dextran in a 2:1 ratio and incubated at 37^o^C for an hour. After gravity sedimentation of erythrocytes the plasma layer containing the remaining blood cells was centrifuged at 450 x g for 7 min and platelet-enriched plasma was separated from leucocyte-containing pellet. Platelets were separated from plasma by centrifugation at 900 x g for 20 min. Leucocytes were re-suspended in 20 mM HEPES buffer pH 7.4, in a 1:1 ratio, layered over Ficoll/Hypaque double gradient media, density, 1.087/1.129 g/ml, and centrifuged at 600 x g for 30 min [11]. Blood mononuclear cells from the upper layer and neutrophils from the bottom layer were collected. Mononuclears were incubated in plastic culture dishes for an hour at 37^o^C with 5% CO_2 _in air, and non-adherent lymphocytes were separated from adherent to the culture plate monocytes. Adherent monocytes were recuperated by gentle scraping with a plastic cell scraper after 15 min incubation with 2.5% disodium-EDTA at 37^o^C.

Bone marrow was flushed from the cavity of long bones (the right and left hind limbs) homogenized in an ice-cold 20 mM HEPES buffer, pH 7.4, filtered through a 70 μm filter to remove bone fragments and a mixture of marrow cells obtained was used in experiments.

Cell preparations from blood and marrow before use were washed twice and re-suspended in 20 mM HEPES buffer, pH 7.4 For the measurement of cellular RNS, L-arginine and L-citrulline content cells were washed twice with PBS, lysed with Triton-X-100, deproteinated with 0.5 N NaOH and 10% ZnSO_4_ or 12.5% TCA. Following a**centrifugation at 900 x g for 5 min**the supernatants were sampled for analysis. Differential cell counts were performed usingsmear technique. Preparations used consisted of about 90 to 95% viable cells as determined with the trypan blue dye exclusion assay.

### NOS Isoforms Assays

The NOS activity was assessed by an accumulation of reactive nitrogen species (RNS), i.e. NO and its stable intermediates (nitrogen oxides, ntrosothiols, etc.) produced by the cells during long-term incubation (at 37^o^C for 22 h) in the incubation medium: 20 mM HEPES buffer pH 7.4, containing 2 mM DTT and 3 mM MgCl_2_∙6H_2_O, (1.3 ml final volume). When incubated in the presence of 1.73 mM CaCl_2_, a total NOS activity of the cells was measured, whilst that iNOS - in the presence of 1.15 mM EDTA (omitting CaCl_2 _from medium); cNOS activity was calculated by subtracting the iNOS activity from the total one. Parallel experiments were conducted, in which samples were incubated in the presence of 5.5 mM L-arginine, and NOS cofactors: 0.2 mM NADPH, 50 μM ((6R)-5,6,7,8-tetrahydro-L-biopterin dihydrochloride) (BH_4_), 5 μM FAD, 5 μM FMN. Reaction was initiated by addition of samples to the incubation medium and terminated by deproteination with 0.5 N NaOH and 10% ZnSO_4_ (as described above). The NOS activity expressed as nmol nitrite (NO_2_^-^) ∙ mg^-1^ protein ∙ 22 h^-1^.

### Reactive Nitrogen Species Measurement

Samples deproteinated with 0.5 N NaOH and 10% ZnSO_4_ were analyzed for the RNS using colorimetric technique based on diazotization reaction detected NO and its derivatives, ntrosothiols, N-nitrosamines, nitrogen oxides (N_2_O_4_, N_2_O_3_, NO_2_^-^, NO_3_^-^) which are presented in the NO solutions even under the best (anaerobic) conditions [12]. Samples were mixed in equal parts with Griess-Ilosvay reagent (1:1 mixture of 0.17% sulfanilic acid and 0.05% α-naphthylamine in 12.5% acetic acid), and 10 min later the diazo-coupled product was analyzed spectrophotometrically at 546 nm. All spectrophotometric absorbance readings for RNS were presented as nitrite and based on measuring with the Griess-Ilosvay reaction dilutions of the stock solution of NaNO_2_.

### L-Arginine and L-Citrulline Measurements

Samples deproteinated with 0.5 N NaOH and 10% ZnSO_4_ were analyzed for L-arginine according to [13] with our modification. Briefly: supernatants were added to a mixture of equal amounts of 0.4 % 8-oxychinoline, 5 % sulphosalicilic acid, 0.01 M glycine buffer and 2.5% NaOH, stirred with 1 % sodium hypobromide (10:1) and 10 min later analyzed spectrophotometrically at 525 nm. Samples deproteinated with 12.5 % TCA were analyzed for L-citrulline using test-system Bio La-Chema (Chechia). The concentrations of L-arginine and L-citrulline were calculated using their measured standards.

Protein concentration was measured according to [14] using crystalline bovine serum albumin as standard.

### Statistical Analysis

Data were expressed as the mean**s** ± S.E.M. and compared using Student’s *t*-test. Relationships between biochemical parameters were determined calculating the Pearson linear correlation coefficient (r). Differences were considered significant at *P* <0.05.

## RESULTS

To determine whether endogenous NO synthesis *in vitro* were detectable, long-term incubation of marrow and blood platelet and leucocyte subpopulations was established. NO and its reactive intermediates production by the cells was determined either in the absence or presence of the exogenous substrate and cofactors of NOS in the incubation medium. N^G^-monomethyl-L-arginine (L-NMMA), which acts as a competitive inhibitor of both constitutiveand inducible isoforms of NOS, at concentration of 2.5 mM, blocked *in vitro* NO generation, suggesting that NO was produced *via *L-arginine NO pathway (data not shown).

### NO Precursors and Metabolites in Blood and Marrow Following Cyclophosphamide Treatment

**Fig. (1):**
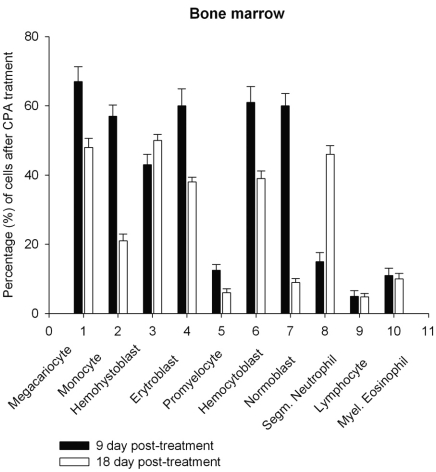
Serp-1 binds to heparin in vitro. In a pull-down experiment with heparin/sepharose beads (A), Serp-1 (100 ng) was incubated with 15 µl of heparin/sepharose beads (Amersham) for one hour at 37 °C. After Serp-1 bound to heparin beads, 100 µl of NaCl solution at different concentrations (0 M, 1 M, and 2 M) was added to elute Serp-1 from the heparin beads. The elution was performed at room temperature for 10 minutes. After elution, the Serp-1 heparin beads were loaded on SDS-PAGE gel for electrophoresis and followed by the immunoblot detection with the anti-Serp-1 monoclonal antibody. 100 ng Serp-1 positive control shown in lane 1. FPLC chromatograph analysis: native (B) and heat-inactivated (C) Serp-1 were loaded onto the heparin columns and eluted with a NaCl gradient. The absorbance at 280 nm was monitored. A representative elution curve is presented from three repeat experiments. X axis at bottom indicates running time of the column analysis. X axis at top indicates collecting fractions (1 ml/fraction). Y axis indicates absorbance at 280 nm. Amino acid sequence alignment (D). Human PAI-1 helix D heparin-binding domain (13 amino acid residues) aligns with the Serp-1 sequence by MacVector (version 3). The residue (Arg6) in Serp-1 has been mutated (boxed) to examine its significance of the heparin binding ability (see text). The identical amino acids in both sequences are dotted and the similar amino acids are starred underneath. 3-D serpin structure showing the relative positions between helix D (hD) and the reactive center loop (RCL) is adapted from Gettins (Chem Rev 2002).

**Fig. (2):**
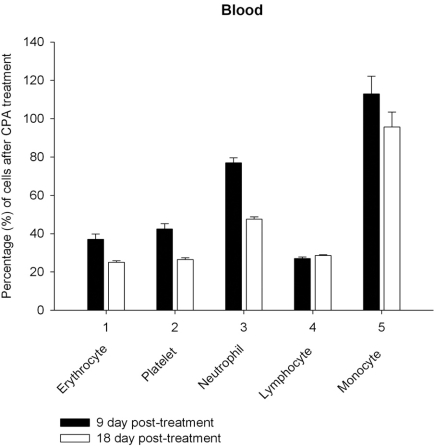
Heparin enhancing effect in the inhibition of thrombin by Serp-1. Thrombin at a fixed amount (0.002 NIH units) was incubated with Serp-1 at various amounts (A-H) in the presence or absence of heparin at 37 °C for one hour. The chromogenic substrate (S-2238) was added into the reaction and color development was monitored every hour for first 6 hours and at 12 and 24 hours thereafter (X axis). The residual thrombin activity (Y axis) was expressed as percentage of the control sample, which contained no Serp-1.

Daily intraperitoneal injection of CPA for 5 days (at the doses described in *Materials and Methodology*) resulted in reduced levels of marrow as well as circulating blood formed elements for up to 18 day *pt* (Figs. **1**, **2**).

**Fig. (3):**
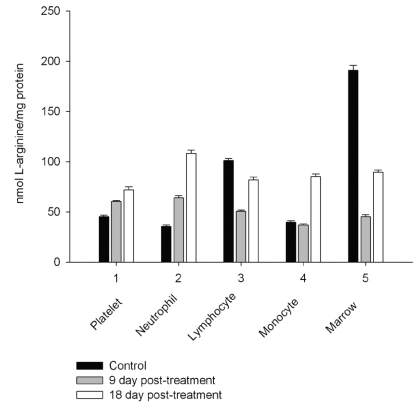
Postoperative radiographic evaluation after two-incision periacetabular osteotomy. Full correction and fixation of the acetabulum was achieved.

**Fig. (4):**
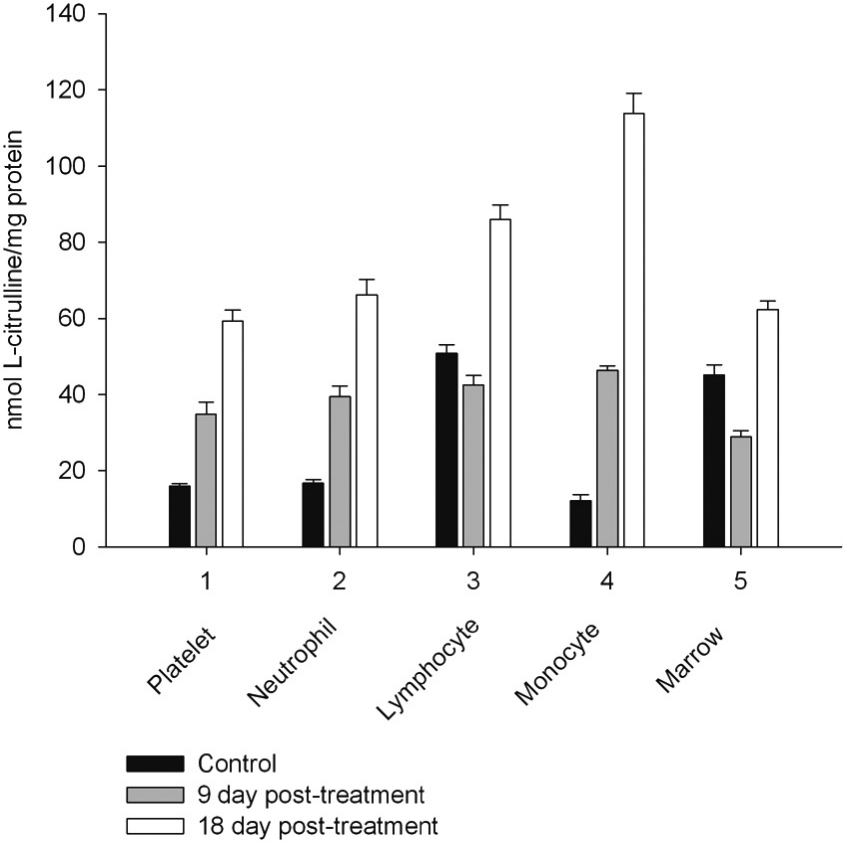
Heparin-dependent inhibition of thrombin by Serp-1. Thrombin at a fixed amount (0.002 NIH units) was incubated with Serp-1 at three non-inhibiting amounts (38, 75, and 150 ng) with increasing units of heparin (X axis). The experiment conditions were similar to those in Fig. 2 except color generation was monitored at 17 hour after the substrate was added into the reactions. The residual thrombin activity (Y axis) was expressed as percentage of the control sample, which contained no Serp-1.

**Fig. (5):**
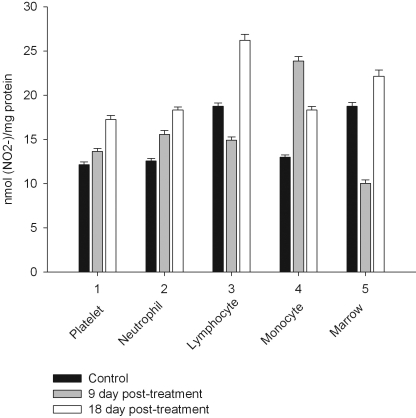
Effect of reactant concentrations of thrombin and heparin in the reaction of Serp-1 and thrombin. 10 µL samples were incubated at room temperature for 20 minutes, followed by adding the SDS-PAGE loading buffer, electrophoresis on a 12% gel, and staining with Coomassie blue kit (Gel-Code Blue, Pierce). Lanes 1 to 8 contained 1 μg Serp-1. Lanes 1 and 9 contained 2 NIH units throm-bin; lane 2, 1 NIH unit thrombin; lane 3, 0.5 NIH units thrombin; lane 4, 0.25 NIH units thrombin; lane 5, 0.12 NIH units thrombin; lane 6, 0.06 NIH units thrombin; lane 7, 0.03 NIH units thrombin. (A) There was no heparin added into the reaction. Heparin at 0.001 IU (B) and 0.01 IU (C) was added prior to the thrombin reaction. S lane contained molecular markers (kDa). Arrows at left indicate the complex band (top) and cleaved Serp-1 band (bottom). Arrowheads at right indicate the native Serp-1 and thrombin bands, respectively.

However, on day 27 most of the animals showed some immune recovery from cytopenia. The cytopenic state of CPA-treated rats might be mimic the clinical situation in immunosuppressive therapy and was accompanied with the changes in the basal cellular levels of NO precursors and metabolites (L-arginine, RNS and citrulline) analyzed as a complementary test of* in vivo *nitrergic response to CPA treatment, and depended on *pt* time. Cellular L-arginine, RNS and L-citrulline contents of platelet, neutrophil and monocyte were gradually increased in 18 days *pt* (with exception for monocyte, in which arginine content was slightly decreased 9 day *pt*), while those of lymphocyte and marrow reduced 9 day *pt* and elevated 18 day *pt* (Figs. **3-5**).

Levels of RNS and L**-**citrulline of marrow, neutrophil, lymphocyte and platelet were highly correlated (r= 0.9864; 0.9876; 0.9978 and 0.9661, respectively, *P*<0.001), to a lesser extent a correlation between those of monocyte was observed (r= 0.3197, *P*<0.001), indicating that NOS reaction metabolites were involved in the other pathways in the latter cells.

### Nitrergic Response to Cyclophosphamide Treatment in Blood and Marrow

NO production by marrow and blood cells from control and CPA-treated rats are shown in Figs. (**6**-**10**). When incubated in the presence of EDTA, all the cells from control rats exhibited the iNOS activity. In the calcium-containing medium, the cells exerted the cNOS activity, with exception for neutrophil and lymphocyte, in which the cNOS was displayed only in the presence of arginine and NOS coactors. The mRNA of iNOS as well as eNOS and nNOS can be reliably detected in the mouse bone marrow [15]. The existence of iNOS mRNA and protein has also been reported for rat neutrophil, eosinophil, megakaryocyte, and unstimulated monocyte, and those of eNOS of lymphocyte, megakaryocyte, and platelet and nNOS of neutrophil [16-18]. It is not excluded that these isoforms contributed to the activity of cNOS and iNOS presented in rat marrow and blood, but strong NOS isoenzymes expression should be further determined at the level of transcription, translation and of enzyme activity to assess NOS isoenzymic spectra of the cells examined. However, under physiological circumstances constitutively active cNOS and iNOS were presented in marrow, as well as blood immune cells, and platelet.

Our results show that marrow and blood cells could produce RNS during long-term incubation even in the absence of L-arginine and NOS cofactors, that on one hand suggest the availability of endogenous arginine for the cells, on the other, that citrulline, co-produced with NO could be converted to arginine *via *the citrulline-NO cycle, which is governed by argininosuccinate synthase and argininosuccinate lyase expressed to some degree in nearly all cell types [reviewed in Ref. 19]. Nevertheless, the requirement for exogenous L-arginine, NAD(P)H, BH_4, _thiols and flavin cofactors for a complete NOS assay system**has been demonstrated, and L-arginine, as well as BH_4_ can promote and/or stabilize the active dimeric form of all three NOS isoforms [reviewed in Ref. 6]. All three NOS isoforms can make superoxide *via* NADPH oxidation at sub-saturated concentration L-arginine and/or BH_4_ [reviewed in Ref. 20, 21]. Notably, a deleterious effect of excessive NO in the tissues is mediated by a potent oxidant, peroxynitite (ONOO^-^, PN), that is readily formed whenever superoxide and NO are produced together [22]. Thus, parallel experiments were performed, where L-arginine and NOS cofactors were included in the incubation mixture to determine *in vitro* a precise total NOS activity and prevent the possible superoxide formation.

Exogenous L-arginine and NOS cofactors increased the iNOS activity of about 1.7 times and that of cNOS only of 1.07 times (compare: in the absence of L-arginine and NOS cofactors the cNOS activity of platelet was of 9 times higher  than that iNOS). When incubated in the presence of L-arginine and NOS cofactors lymphocyte and neutrophil exhibited the cNOS activity, which in neutrophil was of 3.6 times higher, than that of iNOS, while the cNOS activity of lymphocyte was about of 2.5 times lower than that of iNOS. These differences might be related to the differences in the NOS is**o**enzymic spectra of the cells, because cNOS of lymphocyte might be attributed to eNOS (vide supra), while cNOS of neutrophil to nNOS, which mRNA and protein have been shown in freshly isolated rat circulating neutrophils spontaneously producing RNS [23]. The NOS activity was equally distributed between iNOS and cNOS in monocyte, which in the presence of L-arginine and NOS cofactors exerted an elevated activity of iNOS, which prevailed upon the activity of total NOS (determined in the presence of calcium), therefore negative values of cNOS were calculated. It could be speculated that when EDTA was omitted from an incubation medium, exogenous arginine likely stimulate the activity of manganese-dependent arginase there through limiting an availability of arginine for NO synthesis likewise it has been demonstrated in macrophage cultures [24]. However, the RNS production by marrow was diminished in the presence of exogenous L-arginine and NOS cofactors regardless of presence either EDTA or calcium in the incubation medium, indicating that other metabolic processes possibly interfered with the NOS reaction in the cells.

On day 9 after CPA treatment the activities of both cNOS and iNOS were depleted in marrow and blood lymphocyte and accompanied with the impaired levels of arginine, RNS and citrulline of the cells, compared to control. Added L**-**arginine and NOS cofactors influenced negatively the RNS production by marrow, and intensified a drop in the RNS content of lymphocyte incubated in the presence of calcium, but increased it in the presence of EDTA. At the same time, the cNOS activity was manifested in neutrophil (the iNOS was decreased 1, 26 times)and contributed to a remarkable increase in a total NOS activity accompanied with elevated levels of arginine, RNS and citrulline of the cells. Both platelet and monocyte also exhibited enhanced levels of RNS and citrulline, although *in vitro* their RNS production was dropped 9 day *pt*, compared to control. These discrepancies between *in vivo* and *in vitro* testing of nitrergic activity should be further investigated, and may be caused by differences in microenvironment. Added L-arginine and NOS cofactors reinforced a drop in the RNS production by monocyte in the presence either calcium or EDTA 9 day *pt*. Of interest, on 9 day *pt* L-arginine and NOS cofactors in the presence of calcium intensified a decrease in the RNS production by all the cells examined, whereas in the presence of EDTA they attenuated it in platelet and markedly increased the iNOS activity of neutrophil and lymphocyte, once more suggesting a regulatory role of bivalent cations, including calcium, in the effects observed. Our data also suggest that L-arginine and NOS cofactors could alter *in vivo* nitrergic response of marrow and blood to CPA because of competition between NOSs and arginine-metabolizing enzymes and/or NADPH oxidase, possibly stimulated following CPA treatment (vide infra).

On day 18 after CPA treatment an elevated immunosuppression was accompanied with a significant enhancement of arginine, RNS and citrulline content of all the cells studied associated with up**-**regulation of both iNOS and cNOS of blood cells and the iNOS of marrow , compared to control (neutrophil cNOS activity was reduced, but remained markedly higher, than control one). Conversely, the cNOS of marrow was completely suppressed. Of note, the iNOS was increased drastically in platelet, whereas all leucocyte exerted the most pronounced increase in the cNOS activity. Though, the iNOS dominated in these cells with exception for monocyte, in which the cNOS activity was 2.6 times higher, than that of iNOS. Notably, a stimulatory effect of L-arginine and NOS cofactors on the NOS isoforms of platelet and lymphocyte seen in control rats was restored in those of CPA-treated animals 18 day *pt*. At the same time, L-arginine and NOS cofactors depleted the activity of both cNOS and iNOS of neutrophil and that cNOS of monocyte, as well as caused a great decrease in the RNS level of marrow during long-term incubation in the presence of calcium. Contrary, the iNOS of marrow reduced slightly by L-arginine and NOS cofactors, while it was inhibited in control cells.

## DISCUSSION

Our pilot study demonstrates that under physiological circumstances both cNOS and iNOS isoformswere represented in a unique distribution in marrow and blood platelet and leucocyte subpopulations of adult female rat, once more implying a homeostatic role for iNOS. Our data are in agreement with findings that the mentioned NOS isoforms constitutively operate in the immune system [15-18].It should be noted that endogenous NO synthesis *in vitro* assessed byaccumulation of RNS during long-term incubation of cells reflects such counteracted processes, as NO synthesis and its breakdown, and in case of a prevalence of the NO breakdown processes upon its synthesis negative values for the NOS activity are calculated, as for the cNOS (as describrd in *Materials and Methodology*) of control neutrophil and lymphocyte, which RNS production in the presence of calcium was lower, than that in the presence of EDTA. Thus, the NO breakdown processes of these cells are up-regulated (under EDTA omitting conditions) by bivalent cations, particularly calcium. Calcium could trigger a priming of NADPH oxidase in immune cells and enhance superoxide concentration [25, 26] with subsequent formation of PN and impairment of NO. Notably, granulocyte, monocyte and lymphocyte challenged by lipopolysaccharide can produce PN and deplete NO during long-term incubation [27]. Activated human neutrophils cause rapid breakdown of added exogenous NO also due to formation of PN [28]. These processesappear to be activated following CPA treatment (vide infra).

In parallel experiments L-arginine and NOS cofactors combined to either EDTA or calcium in the incubation medium increase the activities of both cNOS and iNOS **in** blood cells (except monocyte cNOS, which is inhibited). Contrary, L-arginine and NOS cofactors cause a remarkable drop of the RNS level of marrow incubated in the presence of either calcium or EDTA (up to negative values). It could be speculated thatadded L-arginine provokes a competition for arginine between NOS and arginine-metabolizing enzymes, such as arginase, argininedecarboxylase (ADC), arginine-specific ADP-ribosyltransferase (ADPRTA) etc, [reviewed in Ref. 19, 29] in the above mentioned cells. Of note, arginase can completely deplete circulating L-arginine thereby significantly reducing the formation of NO [30]. Moreover, iNOS can be inhibited by urea produced in arginase reaction [31]. Pretreatment with arginase inhibitors directly restored NO signalling and arginine responsiveness in aged vessels, as well as in the age-associated decline in cutaneous vasodilatory function [32-34]. Thus, arginasemay limit the available pool of l-arginine for NOS and therefore the bioavailability of NO. Although, the unique and relatively potent inhibition of arginase by the biosynthetic intermediate in NO generation, N^G^-hydroxy-L-arginine suggests an alternate means of regulation in tissues and cells (such as macrophages), that contain both NOS and arginase [35]. Though ADC-derived agmatine is also a competititve inhibitor of the NOS isoforms in macrophages and irreversibly inhibits nNOS and down-regulate iNOS in brain [36, 37]. Thus, cellular NO synthesis rates are regulated *via *a variety of mechanisms that control the availability of arginine [reviewed in Ref. 39]. Undoubtedly, differences in the distribution and expression of the above mentioned enzymes should be contributed to the differences in nitrergic response of blood and marrow.

Hence, the physiological relevance of the *in vitro* testing systems needs to be carefully evaluated, as in the more physiologically relevant condition NO synthesis can exhibit features that do not precisely match the properties of NOS as *in vitro* assessed. A typical example is the `arginine paradox' for NO synthesis, which refers to the dependence of cellular NO production on exogenous L-arginine concentration despite the theoretical saturation of NOS enzymes with intracellular L-arginine [38]. Indeed, in our study *in vitro* tactivation and inhibition of distinct NOSs are highly depended on concentrations of calcium, L-arginine and NOS cofactors, and a chelator agent EDTA, as well. Moreover, the basal levels of NO precursors and metabolites **V** of the cells measured as a complementary test of *in vivo* nitrergic response are not always coincided with a total NOS activity determined in a test tube. Therefore, parallel experiments conducted with L-arginine and NOS cofactors in the NOS assay, as well as determination of basal levels of NOS metabolites of the cells are helpful for clearly understanding of *in vivo* occurred intracellular processes.

The metabolic pattern studied is significantly changed in a CPA-immunosuppressed rat model. CPA-associated myelosuppression, hepatotoxicity and other adverse effects effects are generally known as a response to a reduction in dosage or withdrawal. In our study the capacity of CPA to either activate or suppress the nitrergic response of marrow and blood is changed in a time-dependent manner, possibly related to the retained CPA’s level in the body. Interestingly, immunosuppressive effects of CPA are more pronounced on 18 day *pt*, than 9 day *pt*, indicating to an efficiency of low doses of CPA, which also up-regulate of NOS/NO system of marrow and blood. Of note, on 9 day after the last CPA administration, the inhibition of hematopoesis is accompanied with diminished cellular levels of L-arginine, L-citrulline and RNS (4.2; 1.6 and 1.9 times, respectively), and an impairment of the NO synthesis in marrow, associated with the inhibition of both iNOS and cNOS. The inhibition is reinforced by L-arginine and NOS cofactors, which can affect not only NOS isoforms, but also arginine-metabolizing enzymes, NADPH oxidase etc. Of note, the levels of L-arginine and NO metabolites are significantly higher in both marrow and lymphocyte than in the rest of the cells from control rats, and unlike the latter cells they were decreased in both marrow and lymphocyte *9 day pt*, indicating to utilization of L-arginine *via *other pathways. Though, L-arginine and NOS cofactors increase the iNOS activity of lymphocyte, as well as that of neutrophil 9 day *pt*, suggesting the negative effects of bivalent cations on the NOSs with possible involvement of NADPH oxidase activated by calcium.

Since, a decrease of marrow nitrergic response is suggested by *in vitro* and *in vivo* testing 9 day *pt*, at this stage CPA-induced inhibition of hematopoesis should be provided by other mechanisms. Recently an increased proliferation of neural stem cells has been demonstrated in newborn arginase isoenzyme 1 knockout mice, showing an unanticipated role of arginase in cell growth [39]. Thus, arginase on one hand could inhibit NOS isoforms, on the other prevent cell proliferation. Besides, ADC-derived agmatine can also suppress cell proliferation through an inhibition of ornithine decarboxylase by inducing synthesis of antizyme, a protein that inhibits polyamine biosynthesis and transport [40]. Nevertheless, it should be further investigated whether CPA can activate arginine-metabolizing enzymes, interfering with the NOS reaction, as discussed above.

Enhancedlevel of marrow RNS resulted from the up-regulation of iNOS increased 3.5, times, compared to control, while the cNOS completely suppressed. Contrary to marrow, a gradual increase of neutrophil levels of NOS reaction metabolites are observed resulted from the up-regulation of both iNOS and cNOS that might be involved in the development of granulopenia, and cytopenia in blood 9 and 18 days *pt*. On 9 day after CPA treatment despite of an impairment of the activity of both iNOS and cNOS of platelet and monocyte, the coincident elevation of their citrulline and RNS levels is observed (arginine level of platelet is also increased, whilst that of monocyte is reduced slightly), possibly indicating to an elevation of the NO synthesis. At the same time, L-arginine and NOS cofactors combined to calcium decrease *in vitro* the RNS production by all the cells studied, while combined to EDTA they stimulate the iNOS, with exception for monocyte and marrow. One of the possible explanations of this phenomenon is that on day 9 *pt* CPA induced activation of NADPH oxidase, which in the presence of calcium elevated superoxide production and increased cellular NO breakdown rates in these cells with a subsequent formation PN, which is involved inthe pathogenesis of conditions associated with CPA treatment [41, 42]. CPA has been reported enhance**s** the generation of superoxideby NADPH oxidase [43]. Notably, arginase could also increase superoxide production by myeloid cells through a pathway that likely uses the reductase domain of iNOS [44]. In CPA-administered rats the antioxidant enzymes (superoxide dismutase, catalase, glutathione peroxidase, glutathione-S-transferase, glutathione reductase and glucose-6-phosphate dehydrogenase) showed significantly depressed activities, as well as diminished levels of reduced glutathione, ascorbate and a-tocopherol [45]. In turn, superoxide and H_2_O_2_ contribute to inflammation by induction of intracellular calcium [46]. CPA is shown to affect the ability of heart or liver mitochondria to retain accumulated calcium retention [47].

It should be noted, that direct toxicity of NO *per se* is modest, but greatly increased on reaction with superoxide to form PN [22, 48]. PN is responsible for the T cell-dependent oxidative/nitrative stress facilitated by CPA after bone marrow transplantation [42]. PN anion reacts with biological targets, including CO_2_, in the presence of which nitration yields are increased by 2 to 4-fold [49]. Protonation of PN (pKa of 6.8 at 37^o^C) results in formation of peroxynitrous acid wich is unstable and undergoes either decomposition to nitrate and O_2_ (at pH values ≥ 7.0) or isomerization (predominant at low pH (<7.0)) to nitrate, which thereafter could be reduced to nitrite and NO [50, 51]. These long lasing processes are contributed to the elevated basal RNS level observed, whereas *in vitro* testing shows a drop of the RNS production by platelet and monocyte. Notably, L-arginine and NOS cofactors combined to EDTA increase the iNOS activity of neutrophil and lymphocyte, and attenuate an impairment of the cellular RNS content of platelet during long-term incubation. Thus, PN formation by blood cells could be occurred due to activation of cellular NADPHoxidase in CPA-treated rat**s** 9 day *pt*. Nevertheless, when incubated with EDTA, monocyte exhibit the most remarkable decrease in the RNS level, which reinforced greatly by L-arginine and NOS cofactors, indicating to the involvement of arginine-metabolizing enzymes, as well as other interfering metabolic processes linked to NOS cofactors. L-arginine and NOS cofactors combined to calcium markedly reduce the RNS level of monocyte, neutrophil and marrow during long-term incubation even on day 18 *pt*, that also suggest an opportunity of that kind of scenery.

It is not excluded that CPA-induced elevation of intracellular calcium concentrations trigger an up-regulation of cNOS observed in all leucocyte subpopulations 18 day *pt*, (the cNOS even dominates in monocyte - its activity is 2.6 times higher than that iNOS). However, both iNOS and cNOS are up-regulated by CPA in blood immune cells. Both iNOS and cNOS (nNOS and eNOS) are involved in the compatible regulation of leucocyte recruitment, and iNOS-derived NO inhibits platelet aggregation and platelet and leucocytes adhesion in the microvasculature, modulates cytoskeletal function [reviewed in Ref. 52]. It is of importance, that NO could facilitate down-regulation of intracellular Ca^2+^concentration *via *an activation of either soluble guanylate cyclase *in vivo* and increasing cGMP level [53] or ADPRT A (found in polymorphonuclear leucocyte, lymphocyte and monocyte [54-56]) stimulating mono-ADP-ribosylation of actin in association with inhibition of actin polymerization [53, 57]. Besides, NO in macrophages led to post-transcriptional inhibition of xanthine dehydrogenase/xanthine oxidase, possibly minimizing the potential for tissue injury from XO released from macrophages into the inflammatory milieu [58]. Formation of hydroxylradicals (·OH) by the superoxide-driven fenton reaction can be prevented by NO, which can also attenuate lipid peroxidation [59]. Ameliorating effects of iNOS-derived NO as an antioxidant, antimicrobial and tumoricidal agent has been reported [7, 60]. Thus, NOS/NO system could modulate deleterious side effects of CPA treatment *via *a variety of mechanisms preventing oxidative stress and might be contributed to the attenuation of CPA’s effect on the pro-oxidant system [43]. Finally, NO itself may switch the mechanisms of feedback inhibition of both nNOS and iNOS through formation of inhibitory nitrosyl species [reviewed in Ref. 6]. Recently, a novel negative feedback mechanism has been revealed whereby NO down-regulates iNOS gene expression and NO production by inhibiting the post-translational processes of *IkappaBalpha *there through preventing *NF-kappaB* activation [61]. However, CPA-induced upregulation of NOS isoforms of platelet, especially iNOS (elevated 10.7 times compared to control), could be contributed to overproduction of NO. Of note, iNOS can produce NO for prolonged periods [62]. NO may directly attenuate the activation of platelet, preventing the expression of P-selectin, secretion of platelet granules, intracellular calcium flux, as well as binding of glycoprotein IIb/IIIa to fibrinogen, but could also result in severe detrimental consequences [50], particularly in hemorrhage developed following CPA treatment. A rapid increase in hepatic NO during hemorrhagic shock has been reported [63].

Enhancedlevel of marrow RNS is observed associated with the up-regulation of iNOS increased 3.5, times, compared to control, while the cNOS is completely suppressed 18 days following CPA treatment. CPA-induced down-regulation of cNOS (eNOS and nNOS) may influence the effects driven by these isoforms. nNOS has been shown act**s** as a paracrine effector to regulate hematopoiesis in bone marrow [64]. Recently a role of eNOS in mobilization of stem and progenitor cells has been demonstrated [65]. Nevertheless, the iNOS-derived NO might be involved in the regulation of hematopoiesis as reported for murine granulocyte-containing fractionof marrow, which largely produced NO, there through suppressing cellular proliferation [66]. NO can establish a dominant antiproliferative tone to help protect the stem cell pool from premature depletion when challenged with stressful stimuli [18, 67]. CPA induces the development of early myeloid cells with a potential antitumor effect that may bestrongly antiproliferative through NO production [2]. Of interest, at sub-saturating L-arginine, BH_4_ concentrations, or in the presence of some NOS inhibitors, such as naturally occurring NMMA, iNOS may produce much lower amounts of superoxide than production by nNOS or eNOS [68-70]. We believe that low doses of CPA may be useful in strategies to increase NO availability and antioxidants combined to therapies against NADPH oxidase and other pro-oxidant systems to decrease reactive oxygen species generation there through minimizing deleterious processes associated with several pathological states [71].

## CONCLUSION

Taken together, our results indicate that functionally distinguished Ca(II)/CaM-dependent- and independent NOS isoforms are differentially in a time-dependent manner regulated in blood and marrow in a CPA’s immunosuppressive model of rat. On 9 day after CPA treatment *in vivo* and* in vitro* testing suggest a down-regulation of both cNOS and iNOS in marrow indicating that signals in addition to NO appear to be involved in the inhibitory effect of CPA on hematopoiesis. The most pronounced inhibition of hematopoesis, as well as granulopenia, thrombocytopenia, aplasia, and hemorrhage observed in 18 days *pt* are accompanied with the enhancedlevels of the NOS substrate and metabolites (L-arginine, RNS and L-citrulline) due to up-regulation of iNOS in marrow and blood formed elements (erythrocyte were not sudied). CPA increases thoroughly the cNOS activity of all the leucocyte subpopulations indicating to a possible contribution of the constitutiveisoforms of NOS to the development of CPA’s immunosuppressive effects in blood, which may be also important in the design of therapeuticagents. Contrary, a complete inhibition of marrow cNOS was observed 18 day *pt* that may influence the effects driven by nNOS and eNOS in hematopoesis. L-arginine and NOS cofactors affect *in vitro* the activity of distinct NOS isoforms of marrow and blood platelet and leucocyte subpopulations from control and CPA treated rats suggesting the involvement of L-arginine metabolic pathways, as well as pro-oxidant system, including NADPH oxidase in the regulation of nitrergic response of marrow and blood. Thus, a CPA-induced prolonged cytopenia is accompanied with a time-dependent complex nitrergic response and suggests a complex regulatory network involved inthe pathogenesis of conditions associated with CPA treatment and should be taking into account considering this in the design of chemoimmunotherapysequencing.

## Figures and Tables

**Fig. (6):**
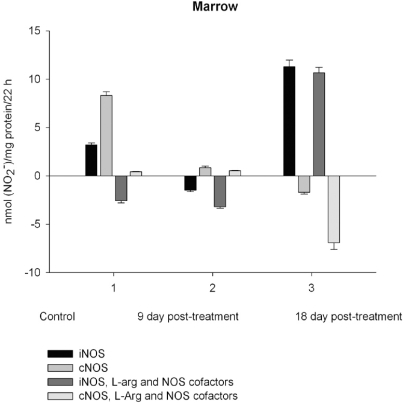
Cleavage of Serp-1 by thrombin in the presence of heparin. (A) The experimental conditions were identical to those in Fig. 5. Lanes 1 to 7 contained 1 µg Serp-1 and 0.03 NIH units thrombin. Lane 8 contained 1 µg Serp-1. Prior to adding thrombin into the reaction, heparin was incubated with Serp-1 at room temperature for 30 minutes. Lane 1, 0 IU heparin; lane 2, 0.0001 IU heparin; lane 3, 0.001 IU heparin; lane 4, 0.005 IU heparin; lane 5, 0.01 IU heparin; lane 6, 0.05 IU heparin; lane 7, 0.1 IU heparin. (B). Protein band density was scanned by the Gel-doc system (Bio-Rad) and plotted as a function of heparin units.

**Fig. (7):**
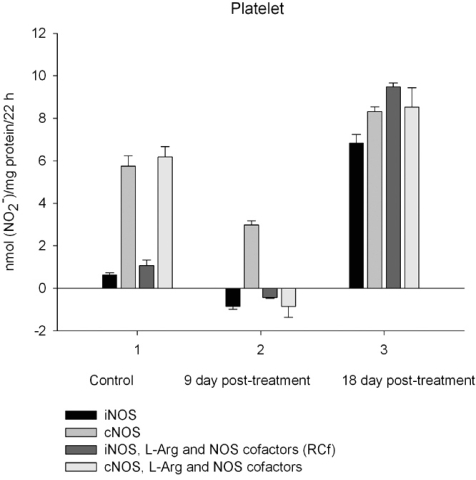
Core membrane fluidity measured using 1,3-Bis-pyrenylpropane (BPP) fluorescent probe. Membrane fluidity in the human monocytic cells (THP-1) was measured after incubation with different activators and inhibitors. Serp-1 (p=0.553), thrombin (p=0.113), or heparin (p=0.158) alone did not cause membrane fluidity changes when compared to control (saline). In the presence of heparin (0.1 U/ml), thrombin increased membrane fluidity sig-nificantly (p<0.001). Serp-1 reversed the heparin-mediated activation, bringing the fluidity level back to a normal level (p < 0.001 compared to heparin activation; p=0.793 on comparison to saline, heparin or thrombin alone without Serp-1). *Significantly different (p<0.001) compared to control saline.

**Fig. (8):**
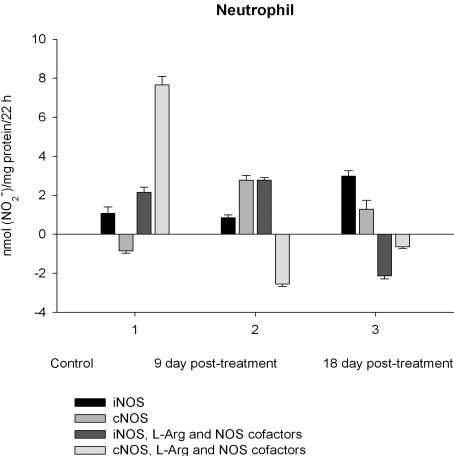
Effect of reactant concentrations of thrombin and heparin in the reaction of Serp-1 and thrombin. 10 µL samples were incubated at room temperature for 20 minutes, followed by adding the SDS-PAGE loading buffer, electrophoresis on a 12% gel, and staining with Coomassie blue kit (Gel-Code Blue, Pierce). Lanes 1 to 8 contained 1 μg Serp-1. Lanes 1 and 9 contained 2 NIH units throm-bin; lane 2, 1 NIH unit thrombin; lane 3, 0.5 NIH units thrombin; lane 4, 0.25 NIH units thrombin; lane 5, 0.12 NIH units thrombin; lane 6, 0.06 NIH units thrombin; lane 7, 0.03 NIH units thrombin. (A) There was no heparin added into the reaction. Heparin at 0.001 IU (B) and 0.01 IU (C) was added prior to the thrombin reaction. S lane contained molecular markers (kDa). Arrows at left indicate the complex band (top) and cleaved Serp-1 band (bottom). Arrowheads at right indicate the native Serp-1 and thrombin bands, respectively.

**Fig. (9):**
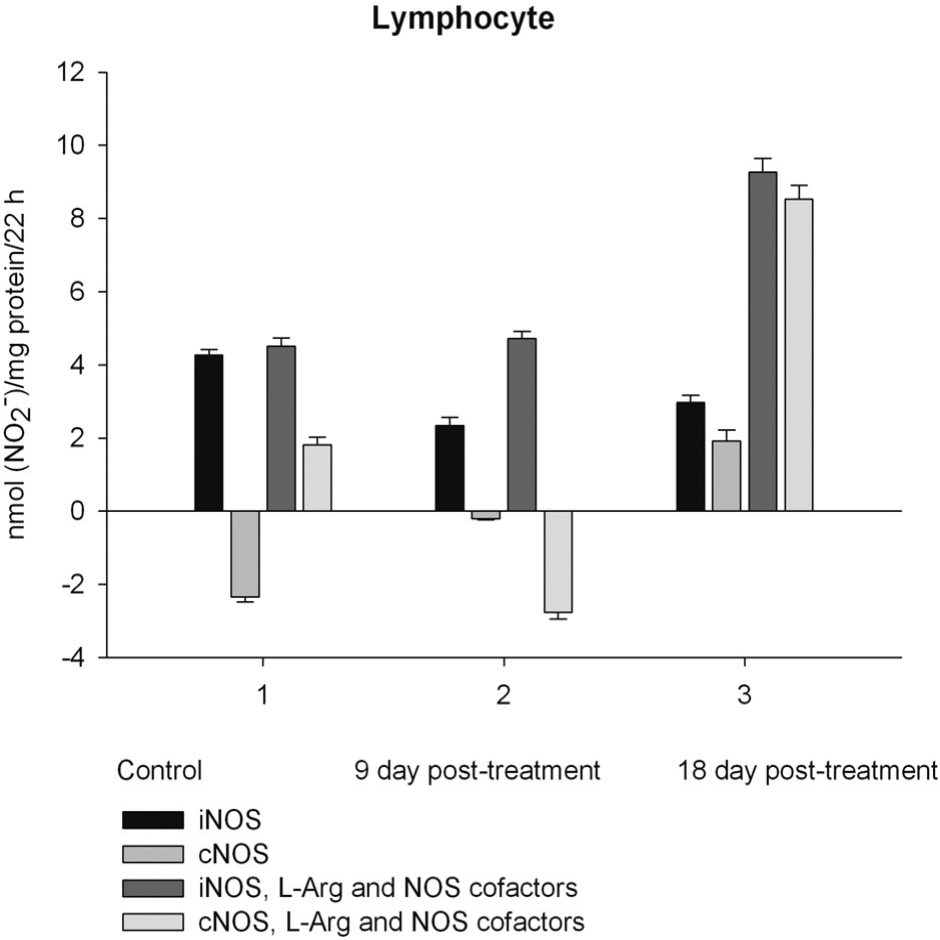
Cleavage of Serp-1 by thrombin in the presence of heparin. (A) The experimental conditions were identical to those in Fig. 5. Lanes 1 to 7 contained 1 µg Serp-1 and 0.03 NIH units thrombin. Lane 8 contained 1 µg Serp-1. Prior to adding thrombin into the reaction, heparin was incubated with Serp-1 at room temperature for 30 minutes. Lane 1, 0 IU heparin; lane 2, 0.0001 IU heparin; lane 3, 0.001 IU heparin; lane 4, 0.005 IU heparin; lane 5, 0.01 IU heparin; lane 6, 0.05 IU heparin; lane 7, 0.1 IU heparin. (B). Protein band density was scanned by the Gel-doc system (Bio-Rad) and plotted as a function of heparin units.

**Fig. (10):**
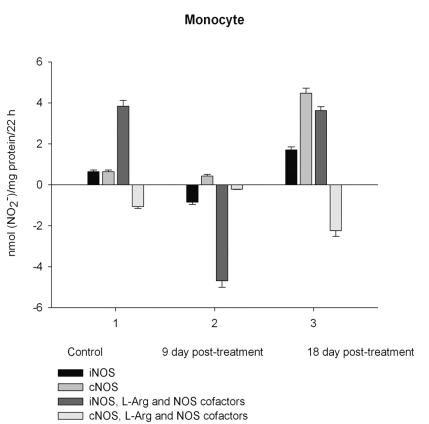
Core membrane fluidity measured using 1,3-Bis-pyrenylpropane (BPP) fluorescent probe. Membrane fluidity in the human monocytic cells (THP-1) was measured after incubation with different activators and inhibitors. Serp-1 (p=0.553), thrombin (p=0.113), or heparin (p=0.158) alone did not cause membrane fluidity changes when compared to control (saline). In the presence of heparin (0.1 U/ml), thrombin increased membrane fluidity sig-nificantly (p<0.001). Serp-1 reversed the heparin-mediated activation, bringing the fluidity level back to a normal level (p < 0.001 compared to heparin activation; p=0.793 on comparison to saline, heparin or thrombin alone without Serp-1). *Significantly different (p<0.001) compared to control saline.
